# Retraction: Pyrophosphate modulates plant stress responses via SUMOylation

**DOI:** 10.7554/eLife.46993

**Published:** 2019-03-26

**Authors:** M Görkem Patir Nebioglu, Zaida Andrés, Melanie Krebs, Fabian Fink, Katarzyna Drzewicka, Nicolas Stankovic-Valentin, Shoji Segami, Sebastian Schuck, Michael Büttner, Rüdiger Hell, Masayoshi Maeshima, Frauke Melchior, Karin Schumacher

Görkem Patir-Nebioglu M, Andrés Z, Krebs M, Fink F, Drzewicka K, Stankovic-Valentin N, Segami S, Schuck S, Büttner M, Hell R, Maeshima M, Melchior F, and Schumacher K. 2019. Pyrophosphate modulates plant stress responses via SUMOylation. *eLife* **8**:e44213. doi: 10.7554/eLife.44213.

Published 20, February 2019

The authors are retracting the *eLife* paper cited above based on irregularities associated with the western blots shown in Figures 3D and E as well as Figures 4C and D performed by Gorkem Patir-Nebioglu (GPN). Initiated by reports on PubPeer, the authors have investigated the matter and have found that in both Figures 3 and 4 the blots were spliced together from different original images without indication and a single lane was copied twice in Figure 3E. In addition, GPN has admitted to having intentionally loaded less protein for fugu5 at 4°C (Figure 3) and 40°C (Figure 4). The authors are currently running independent repetitions of the experiments in question and hope to be able to provide appropriate data in the near future. However, with the currently available data, the conclusion that SUMOylation is reduced in fugu5 mutants is not justified and all authors have agreed to retract the paper.

